# A Psittacosis Outbreak among English Office Workers with Little or No Contact with Birds, August 2015

**DOI:** 10.1371/currents.outbreaks.b646c3bb2b4f0e3397183f31823bbca6

**Published:** 2018-04-27

**Authors:** John Mair-Jenkins, Tracey Lamming, Andy Dziadosz, Daniel Flecknoe, Thomas Stubington, Massimo Mentasti, Peter Muir, Philip Monk

**Affiliations:** Field Epidemiology Training Programme, Public Health England, Nottingham, United Kingdom; European Programme for Intervention Epidemiology Training (EPIET), European Centre for Disease Prevention and Control (ECDC), Stockholm, Sweden; Field Epidemiology Service, National Infection Service, Public Health England, Nottingham, United Kingdom; Public Health England East Midlands, Nottingham, United Kingdom; Environmental Health, Ashfield District Council, United Kingdom, United Kingdom; Public Health England East Midlands, Nottingham, United Kingdom; Public Health England Midlands, Nottingham, United Kingdom; Bacteriology Reference Department, Respiratory and Vaccine Preventable Bacteria Reference Unit, Public Health England, London, United Kingdom; Public Health Laboratory Bristol, National Infection Service, Public Health England, Bristol, United Kingdom; Public Health England East Midlands, Nottingham, United Kingdom

## Abstract

**Introduction:**

On 14th August 2015 an office manager informed Public Health England of five employees known to have been diagnosed with pneumonia over the previous three weeks. We investigated to establish whether an outbreak occurred and to identify and control the source of infection.

**Methods:**

We undertook case finding for self-reported pneumonia cases at local businesses (July-August 2015). Clinical samples from a hospitalised case were tested for common respiratory pathogens, but returned negative results. Further testing confirmed *Chlamydia psittaci* infection in this case (serology and PCR).  We subsequently undertook* C. psittaci* testing for all cases, redefining them as confirmed (*C. psittaci* PCR or high antibody titre via serology) or probable (inconclusive *C. psittaci* serology). Twenty-eight day exposure histories informed descriptive epidemiological analysis. We conducted an environmental investigation at the office to identify potential sources of exposure.

**Results:**

We identified six office workers with pneumonia; four met case definitions (three confirmed, one probable) with symptom onset between 29th July and 4th August 2015. Workplace was the only epidemiological link and only one case reported limited, indirect bird contact. Environmental investigations identified pigeons roosting near the office which were being fed by workers (none cases).

**Discussion:**

This was a probable outbreak of psittacosis with no direct bird-to-human contact reported. Cases recovered after receiving appropriate antibiotics. Feeding of pigeons was stopped. A deep clean of office ventilation systems was conducted and workers were advised to avoid bird contact.  We hypothesised that indirect environmental exposure to infected pigeons was to the source of this outbreak. This work provides evidence that health professionals should consider psittacosis in the differential diagnosis of cases of severe or atypical respiratory illness even without overt bird contact.

## Introduction

On the 14th August 2015 an office manager contacted Public Health England (PHE) after becoming concerned about five employees who had been diagnosed with pneumonia within the previous three weeks. All worked in the same modern, open-plan office which was part of a small industrial estate with three occupied buildings. PHE initiated an investigation, suspecting a possible workplace exposure to* Legionella*^1^^,^^2^.

Initial investigations identified three employees who were admitted to different hospitals and clinicians treated all cases as community acquired pneumonia (CAP) leading to limited microbiological testing and empirical treatment. A lack of microbiological diagnosis meant these cases had not been notified to PHE through routine surveillance systems, and an epidemiological link between cases had not been established. An interview with the office manager established that there had previously been problems with pigeons nesting on the building and in the internal roof space. These issues had been resolved several months prior to the outbreak, however *Chlamydia psittaci* was subsequently included as a potential causal organism and psittacosis among our list of initial differential diagnoses.

Human psittacosis may be asymptomatic , cause a mild influenza-like illness, pneumonia, or occasionally myocarditis, hepatitis or encephalitis^3^. It is unlikely to be suspected by clinicians unless bird contact is reported and is rarely diagnosed, with only 26 laboratory confirmed *C. psittaci* infections reported through routine surveillance in England in 2014^4^. Psittacine birds (parrots, parakeets and macaws) and pigeons are the main hosts shedding infectious particles in faeces and respiratory secretions facilitating zoonotic transmission by dried guano or dust from feathers^5^. As such most psittacosis cases and outbreaks are associated with occupational or pet bird contact^6^^,^^7^, although transient or indirect exposure to infected birds, guano or dried excretions have been associated with infection^8^^,^^9^.

Here we report the findings of the multiagency incident control team investigation into a cluster of human psittacosis cases, and the impact of delayed diagnoses on the public health investigations.

## Methods

**Ethics Statement**

This study represents the findings of a public health investigation into a suspected infectious disease outbreak. The investigation was carried out by Public Health England and the Local Authority within the statutory framework for public health in the UK. Therefore, the investigation was not subject to ethics approval. Investigators had access to patient identifiable data to facilitate investigations and provision or public health advice. Public Health England base our use of information on adherence to the UK Data Protection Act 1998, the law for notifiable disease and section 251 of the NHS Act 2006 (originally enacted under Section 60 of the Health and Social Care Act 2001). Data relating to infectious disease outbreaks are therefore processed legally without consent under Regulation 3di of section 251 of the NHS Act 2006 (http://www.legislation.gov.uk/uksi/2002/1438/regulation/3/made). Verbal consent for each patient was collected by clinicians in the public health team who were following up cases. It was recorded in the clinical records held for each case as part of the public health investigation.

**Epidemiological investigation**

We used staff sickness records and worked with office managers of all three occupied office buildings to undertake case finding at the businesses. Initial case definitions were designed to identify cases of self-reported pneumonia or lower respiratory tract infection (LRTI) (confirmed from medical records) working in or visiting the office or adjacent buildings from July 2015 onward.

Once microbiological results established a diagnosis of psittacosis we supplemented case finding with searches of statutory clinical and laboratory notification data and updated our case definitions. We defined confirmed cases as those with a diagnosis of pneumonia or LRTI, working in or visiting the office or adjacent buildings, between 1 July and 1 September 2015 with PCR confirmed *C. psittaci* infection or *C. psittaci* antibody titre consistent with infection (defined as 1:512 for the purposes of this investigation). Probable cases were those meeting the criteria above without positive PCR results and inconclusive serological results.

PHE staff interviewed cases or family members using a piloted questionnaire to collect details of symptoms, clinical testing and treatment, household and work contacts and a travel history, as well as details of potential exposures in the 14 days prior to onset of symptoms. This time frame included the incubation periods of most common causes of CAP and Legionnaires’ disease^10^. We re-interviewed cases once a diagnosis of psittacosis was established to collect additional clinical information and identify bird, feather, guano or animal exposures in the 28 days prior to symptom onset. We used this information along with staff attendance records to inform descriptive analyses.

**Microbiological investigations**

At the outset of this investigation there was only one hospitalised patient able to provide clinical samples. Routine testing for common respiratory pathogens was carried out, along with real-time PCR testing for* C. psittaci *11 and* Legionella* and serological testing for *C. psittaci* at Public Health England (accredited laboratories).

Once a diagnosis of psittacosis was established, we requested *C. psittaci* paired sera for all potential cases. Serological testing used a two-step approach with samples initially tested using a *Chlamydia* group reactive complement fixation test (CFT) and if positive (a titre≥8 is consistent with recent *Chlamydia/Chlamydophila* species infection) they were further tested for *C. psittaci /C. abortus* antibodies using an indirect immunofluorescent antibody (IIF) assay. The IIF test employed an in-house assay using enzootic abortion of ewes (EAE)-infected McCoy cells as antigen, and a combined anti-human IgG/M/A fluorescein conjugate (Inova Diagnostics).

Diagnosing psittacosis using serological testing alone is challenging due to a risk of cross reaction between *C. psittaci* and Chlamydia pneumoniae antibodies. Within the context of this investigation assays with positive CFT results and a high IIF titre (> 1:256) were interpreted as providing evidence consistent with *C. psittaci* infection. Assays with lower IIF titres, but showing reactivity were described as providing evidence of possible infection. No further sputum samples were available and typing of *C. psittaci* was not undertaken.

**Environmental investigations**

Investigations by Environmental Health Officers initially focused on potential sources of exposure to *Legionella* and then for potential sources or routes of zoonotic transmission after a diagnosis of psittacosis was established. This investigation included mapping the internal layout of the office building, the desk locations of potential cases and the design of the ventilation system. We also looked for potential roosting sites of birds and at the extent of the pigeon and other wild bird populations in the local area and inspected devices installed at the office building to prevent bird infiltration.

## Results

There was a 30 day delay in diagnosis of psittacosis after onset of symptoms due to low clinical suspicion and no history of direct bird contact reported by cases. During this time six office workers with LRTI were identified (from two office buildings). Once psittacosis was diagnosed only four office workers met revised case definitions (three confirmed, one probable), all worked in one office. Cases had a median age of 49 years (range 43-63 years) and 50% (2/4) were female.

All cases reported symptoms consistent with psittacosis (Table 1) with onset from 29th July 2015 to 4th August 2015 (Figure 1). The possible case was diagnosed and treated within the community; however the three confirmed cases had more severe symptoms requiring hospitalisation. One of these cases was admitted for 41 days, 35 of which were spent ventilated on an intensive treatment unit (Table 1).

**Table 1: Diagnostic data for and background information for psittacosis cases by date of onset, England, August 2015 (n=4):** *Testing of historic sample. This PCR assay cannot differentiate between* C. psittaci* and *C. abortus*. CAP: Community acquired pneumonia, LRTI: Lower respiratory tract infection, Case 1,2,4 met confirmed case definition, Case 3 met probable case definition. N/A: not available

Case	Clinical diagnosis and reported symptoms	Symptom onset	Hospital admission	Length of stay	PCR (sputum date)	Serology Titre (sample date)		
1	CAP, fever, nausea/vomiting, myalgia, cough	29/07/2015	10/08/2015	41 (35 days ventilation)	Positive* (13/08/2015)	2048 (1/08/2015), 2048 (28/08/2015)		
2	CAP, fever, nausea/vomiting, myalgia, dizziness, cough, headache	01/08/2015	04/08/2015	3	N/A	512 (04/09/2015)		
3	CAP, fever, nausea/vomiting, dizziness, cough, headache, abdominal pain	03/08/2015	Treated in community	N/A	N/A	256 (24/09/2015)		
4	LRTI, fever, nausea/vomiting, myalgia, dizziness, diarrhoea	04/08/2015	13/08/2015	4	N/A	512 (03/09/2015), 512 (04/09/2015)		

**Onset dates of symptoms and chronology of investigation following an outbreak of psittacosis in an office in England, July- September 2015:**
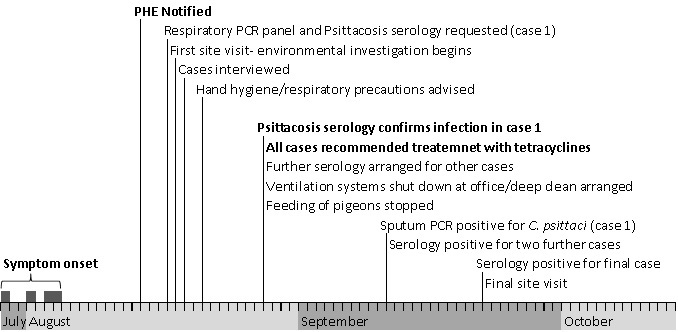

**Epidemiological links and potential exposures**

Following identification of psittacosis cases were asked explicitly about exposures to birds, feathers or guano. Case 1 (index) reported indirect contact at his home when disposing of a dead pigeon, using a long-handled garden tool, approximately one week before onset of symptoms. The three other cases reported no contact with birds, feathers or guano in the 28 days prior to onset at work or anywhere else.

Cases did not live close to one another, did not socialise together, and comparison of travel histories did not identify any commonalities and workplace was the only epidemiological link identified. Two cases (Case 1 and 4, Table 1) reported sitting at nearby desks and working together in the office, but otherwise there were no reports of close contact at work. Attendance records identified only four days between 13 July and 23 July 2015, during the incubation period, when the four cases were present together at the office, suggesting a ten day window of exposure. Estimates of the incubation period in this outbreak ranged from a median of 10 days (range 6-12 days from the latest date in the exposure window), up to a median of 21 days (range 17-23 days from the earliest date in the exposure window).

**Microbiological and serological findings**

Routine testing for CAP failed to identify a causal organism, however serological testing for Chlamydia group and *C. psittaci* from Case 1 identified infection which was confirmed by *C. psittaci* PCR testing of a historic sputum sample (Table 1). Serological testing of Cases 2-4 was undertaken, however due to delays in diagnosis sputum samples were not available. These cases had been discharged and were being treated in the community, hampering the collection of paired sera.

Where only one sample was available or there was a short time between samples (cases 2 and 4) the results were interpreted as providing evidence of recent *C. psittaci* infection due to the high titre (1:512). Case 3 had a titre from a single sample which suggested possible infection but further samples were not available. Due to the risk of antibody cross reactivity, infection with C. pneumoniae in cases 2-4 could not be ruled out.

**Environmental investigation and site visits**

The environmental investigation at the site did not identify any specific issues with the office building. Several months prior to the first psittacosis case pigeons had infiltrated the internal roof space. Netting and anti-bird devices had been installed on the office roof at this time (prior to incubation period) and appeared to be effective after visual inspection.

The building ventilation system had two separate external air inlets located on the roof suggesting a potential zoonotic transmission pathway, however anti-bird netting was in place over inlet areas and there was no evidence of nesting or infiltration of birds (e.g. guano or feathers) near inlets. Mapping of ventilation systems also showed that cases were seated in different areas of the office fed by separate inlets on the roof. Ventilation systems in the office were shut down as a precaution during the investigation, and the system underwent a deep clean and all air filters were changed. It was not possible to collect environmental samples from the ventilation system prior to it being cleaned.

During the site visit it was reported that workers from nearby offices (non-cases) often fed the birds which had attracted large numbers of pigeons. This had caused historical problems with guano on the pavements surrounding the office to the extent where cleaning of pavements was undertaken. This prevented environmental sampling of bird guano from around the building. The car park was adjacent to the office, but the staff did not report a particular problem of guano on cars, and no cases reported contact with guano. Feeding of birds was subsequently stopped.

We worked closely with the affected office to communicate the steps and results of the investigation. Office staff were provided advice about respiratory precautions, and avoiding contact with birds, guano and feathers and we continued to actively monitor staff sickness reports for several months to ensure no further cases developed.

## Discussion

Our investigation identified a probable outbreak of psittacosis epidemiologically linked to an office where problems with pigeons had previously been reported. We identified a PCR positive psittacosis case, two further cases had high antibody titres and the final case had moderate titres. The available evidence suggested psittacosis as the most likely diagnosis and the investigation and public health actions were based on this. No cases reported direct exposure to birds and we hypothesised that pigeons roosting near the office were the source of infection. However, we were not able to establish microbiological link between birds and cases. Feral pigeon populations have been previously been linked with psittacosis and regular feeding of pigeons , as reported here, is implicated in the establishment of large feral pigeon populations^12^.

Cases may have been exposed outside the office and we also investigated whether indoor exposure may have occurred via the ventilation system. The environmental investigation found no visual evidence of contamination in the ventilation systems and all inlets were protected from bird ingress, however we were unable to collect environmental samples and could not rule out this potential pathway. Indirect transmission from birds in the area outside of the office on one or more of the days in mid-July when all cases were working, may have been more feasible. The exact nature of the zoonotic transmission event remains unclear, however this hypothesis is supported in the wider literature. For example aerosolisation of infectious particles by lawn mowers^13^ or cleaning of bird feeders have been previously implicated^14^. There is also growing evidence of outbreaks with no direct link to birds^15^, and it has been suggested that human psittacosis may be endemic in some areas where environmental exposure to birds is common^16^.

Person-to-person transmission from the index case who reported indirect contact with a dead pigeon was considered, but ruled out. This was because of insufficient close contact to explain all cases and serial intervals of less than the reported minimum seven day incubation period^10^. In addition, person-to-person transmission has rarely been reported with recent reports generally linked with nosocomial exposure^17^.

**Limitations**

The delay in recognition of this cluster led to limited availability of clinical samples to enable the definitive diagnosis of *C.psittaci*. This led to diagnoses based on serology in three cases which were at risk of cross reaction with *C.pneumoniae* and therefore this could not be ruled out as an aetiology. However the severity of reported symptoms and the lack of laryngitis in any cases (commonly associated with *C.pneumoniae*) suggest psittacosis as a likely aetiology. The delay also impacted on the collection of paired sera as three of the cases were in the community by the time of investigation.

Actions in this investigation were based on psittacosis as the most likely diagnosis, given the epidemiological links between cases, PCR and serology results. However, it would have been desirable to have further samples to enable testing and further subtyping, along with samples from pigeons or guano in the environment to allow comparison. Serological testing of non-cases working in the same environment may also have been useful in determining the clinical attack rate, as positive psittacosis serology is a relatively unusual finding. The delay in diagnosis also resulted in an extended recall period, which may have impacted on the accuracy and completeness of the epidemiological information gathered.

**Implications for Public Health**

Outbreaks of psittacosis are relatively rare and this investigation highlighted practical problems encountered when delayed diagnosis limits the available samples to provide a definitive diagnosis. This investigation adds to evidence of human psittacosis cases where no direct link or close contact with birds is reported and suggested a median incubation period of between 10-21 days.

Psittacosis is not routinely included in the list of differential diagnoses for CAP and this investigation has highlighted that delays in diagnosis may lead to suboptimal antibiotic treatment^18^, prolonged hospital admissions and periods of ventilation for patients with associated high health care costs, and negatively impact public health surveillance and outbreak response^19^. A standard protocol for investigating unexplained outbreaks of pneumonia may help to avoid some of the limitations of this investigation in the future. However, there remains a need for clinicians to be aware of the possibility of human psittacosis in severe LRTIs even when little or no bird contact is reported and the need to expand routine testing to include psittacosis in patients with atypical pneumonia.

This investigation was prompted solely by the report of unusual illness by an office manager. This highlights that active engagement between public health services and local communities is important to help ensure unusual health events are reported. This study also demonstrates a need for increased public awareness of the risk of psittacosis in people who spend time with birds and we recommend feeding of feral pigeons in populated or commonly used areas should be prohibited^8^.

**Conclusions**

This investigation into a probable outbreak provides evidence of transmission of psittacosis in one or more office workers with no direct bird contact. In light of this clinicians and public health professionals should be aware of the possibility of psittacosis in cases of severe respiratory illness reporting no overt bird contact and consider it on their list of differential diagnoses in situations where indirect exposure to birds is possible, but not necessarily obvious.

## Competing Interests

The authors have declared that no competing interests exist.

## Corresponding Author

John Mair-Jenkins: john.mairjenkins@phe.gov.uk

## Data Availability

The data underlying the findings of this study contain potentially identifying information of the study participants and cannot be de-identified. Therefore, in adherence to UK legislation enacted by Public Health England the data is only available upon request. Requests for data may be sent to Public Health England (FOI@phe.gov.uk). All other relevant data may be found within the paper.
